# Networks of inflammation, depression, and cognition in aging males and females

**DOI:** 10.1007/s40520-022-02198-6

**Published:** 2022-07-27

**Authors:** Rebecca A. Chalmers, Matti Cervin, Carol Choo, Bernhard T. Baune, Julian N. Trollor, Katya Numbers, Perminder S. Sachdev, Henry Brodaty, Nicole A. Kochan, Oleg N. Medvedev

**Affiliations:** 1 University of Waikato, Hamilton, New Zealand; 2 Lund University, Lund, Sweden; 3 College of Healthcare Sciences, Division of Tropical Health and Medicine, James Cook University, Townsville, QLD Australia; 4 Department of Psychiatry, University of Muenster, Munster, Germany; 5 Department of Psychiatry, Melbourne Medical School, The University of Melbourne, Melbourne, VIC Australia; 6 The Florey Institute of Neuroscience and Mental Health, The University of Melbourne, Parkville, VIC Australia; 7 Centre for Healthy Brain Ageing, School of Psychiatry, UNSW Sydney, Kensington, NSW Australia; 8 Department of Developmental Disability Neuropsychiatry, School of Psychiatry, UNSW Sydney, Kensington, NSW Australia; 9 Neuropsychiatric Institute, The Prince of Wales Hospital, Randwick, NSW Australia

**Keywords:** Biomarkers, Cognition, Depression, Aging, Network analysis

## Abstract

**Background:**

Prioritizing the maintenance of healthy cognitive aging and personalizing preventive interventions to enhance their effectiveness is crucial as the global population ages. Systemic inflammation and depression in older people have been associated with decreased levels of cognition but results have been inconsistent.

**Aims:**

To explore the interactive network of inflammation, depression and cognition by sex in older people.

**Methods:**

We used novel network analysis to explore the unique associations between inflammatory biomarkers, depression, cognition, and somatic, genetic, and lifestyle risk factors in an older (aged 70–90 years), non-demented, community-dwelling sample from the longitudinal Sydney Memory and Aging Study (*N* = 916) at baseline and at a two-year follow-up.

**Results:**

The networks of biomarkers, depression, cognition, and relevant covariates were significantly different between males and females. A stable negative link between depression and cognition was found in females only; a stable positive association between biomarker interleukin-6 and depression was found in females only; and a stable positive association between biomarker interleukin-8 and alcohol was found in females only. For both males and females, a stable, positive relationship was found between the presence of APOE-ε4 gene and biomarker C-reactive protein; between education and cognition; and between biomarker interleukin-6 and all other biomarkers.

**Conclusions:**

These findings suggest different psychophysiological mechanisms underlie the interactive network of biomarkers, depression and cognition in males and females that should be considered when designing personalized preventive interventions to maintain cognitively healthy aging.

**Supplementary Information:**

The online version contains supplementary material available at 10.1007/s40520-022-02198-6.

## Introduction

Safeguarding our mental health is crucial for maintaining independent living as we get older [1]. Healthy aging is not only influenced by modifiable lifestyle factors like diet and exercise, but also by biological factors such as sex [2, 3]. Sex differences affect our immune functioning and subsequent inflammation levels [4]. As we age, the risk of developing chronic low-grade systemic inflammation (“inflammaging”) increases [5]. Inflammaging has been associated with higher levels of depression and cognitive impairment in older adults [6, 7]. Sex differences have been found in depression and across cognitive domains in older adults [8, 9]. In light of this research, the relationship between inflammatory biomarkers, depression, and cognitive function in older adults is likely to differ by sex but no studies have specifically examined this in one network. By understanding how inflammation, depression and cognition interact as a function of sex, we can develop interventions to promote healthy mental aging with greater precision and responsivity.

In healthy older populations biological markers of inflammation have been associated with decreased cognitive ability. For example, Baune et al. [10] found that inflammatory biomarker interleukin-8 was associated with poorer memory, cognitive speed, and motor function; Trollor et al. [7] found a significant negative association between inflammatory biomarker interleukin-12 and processing speed, but in females only. Inflammatory biomarkers have also been associated with depressive symptoms in healthy older populations. For example, Baune et al. [6] demonstrated that baseline levels of inflammatory biomarkers interleukin-6 and interleukin-8 were significantly associated with higher levels of depressive symptoms, and that interleukin-8 was also a marker of future development of depressive symptoms. Other studies have found sex differences in the relationship between inflammaging and depression, where inflammation predicted higher depressive symptoms in females but not males, and higher depressive symptoms predicted higher inflammation in males but not females [11].

However, the nature of the depression-cognition relationship in older populations is unclear. Depression can be considered either a risk factor for, or a symptom of, declining cognition, with inflammation being a possible common underlying mechanism for the relationship [6, 12, 13]. Thus, whilst the inflammation-cognition, inflammation-depression and depression-cognition relationships in older adults have previously been explored across cohorts, these relationships are inconsistent and have yet to be examined as a single inter-related system [7, 10, 13, 14]. Exploring the interactive relationship between biomarkers of inflammation, depression, cognition, somatic, genetic, and lifestyle risk factors (e.g., cardiovascular risk, APOE-ε4, alcohol consumption, education) simultaneously using network analysis will enable us to identify factors that have a direct relationship with depression and cognition in an older sample. Further analysing the networks by sex will allow us to understand how these relationships differ in males and females.

The application of network analysis to the understanding of psychopathology is relatively recent; conceptually, network analysis regards psychopathology as a combination of causal and bidirectional relationships between psychological, biological, and environmental factors [15]. Network analysis estimates the strength of relationships between different elements (“nodes”) in a complex system, as well as the importance of each node to the full system (“the network”) [16]. In psychopathology, the nodes are the symptoms—represented by circles—that are joined by coloured lines (“edges”) that connect them [16]. The importance of each node to the network is determined by both the number and strength of its unique associations with other nodes and how much variance in each node can be explained by variation in its neighbouring nodes [17]. Unlike conventional statistical analysis, network analysis includes all relevant variables in a single network to clarify relationships without arbitrarily assigning dependent and independent variables [18]. The advantage of network analysis compared to mediation and moderation analyses and/or linear regressions is that it allows us to look at multiple interactions across several variables simultaneously, reducing the probability of finding false positives due to repeated testing [18].

To date, network analysis has been used to determine relationships between inflammatory biomarkers and depression in non-elderly clinical and population samples. Relationships were found between C-reactive protein and depressive symptoms, including changes in appetite and fatigue, though sex differences were not specifically examined [19–21]. Network analysis has not yet been used to determine relationships between inflammatory biomarkers and cognition or between inflammatory biomarkers, cognition, and depression in older populations.

Biomarkers of inflammation include cytokines, which are the chemical messengers of the immune system that mediate the inflammatory response when one faces an environmental or psychological challenge (e.g., a viral or bacterial attack, injury, or stress) [14, 22]. Under normal conditions without immune challenge (physiological conditions), cytokines are integral to the process of learning, memory, neural plasticity and neurogenesis [23]. However, the delicate balance of neural and immune activity under physiological conditions can be disrupted by inflammaging. Inflammaging is indicated by an overproduction of cytokines when there is no challenge to immunity and has been associated with neuropsychiatric disorders (e.g., major depression) and neurodegenerative disease (e.g., dementia) [23, 24]. Thus, cytokine activity in older populations may play an important role in promoting better cognitive and mental health due to their potential capacity to influence inflammation [24].

The aim of this study was to investigate the interactive network of inflammatory biomarkers, depression, cognition, and somatic, genetic, and lifestyle risk factors in an aging population of males and females over time using network analysis. To do so, we considered the following markers of systemic inflammation: C-reactive protein (CRP) and interleukins (IL)-6, -8, -10, and -12 given their previous associations with cognitive and affective outcomes [6, 7, 14, 25–27]. We also considered the influence of other variables known to impact cognitive function such as education level [28], alcohol consumption [29], the presence of apolipoprotein E4 gene (APOE-ε4) [30] and risk of cardiovascular disease [31]. These links are thought to vary by sex (except education [32]): females develop alcohol related health consequences (including cognitive impact) earlier than males [33]; combinations of APOE-ε4 alleles affect the risk and rate of development of cognitive decline across sexes, but cognitive impact is similar [34]; and exposure to cardiovascular disease risk is greater in older females [35]. We expected that there would be differential associations between biomarkers, cognition, and depression for males and females. Firstly, and uniquely, we used data to examine the overall system of biomarkers, depression, and cognition separately for the males and females in our sample. We then tested whether the major findings were replicated using data collected 2 years later. The division into exploratory and confirmatory analyses strengthens confidence in and replicability of findings.

## Methods

### Participants

In 2005, 8914 community-dwelling, 70–90-year-olds were chosen randomly from the electoral register in Sydney’s Eastern Suburbs (Australia) and invited to join the Sydney Memory and Ageing Study (MAS) [36]. Participants needed sufficient English to complete psychometric assessments, self-report questionnaires and informed consent. Exclusion criteria included prior dementia diagnosis, major psychological or neurological disorder, or progressive malignancy. Participants with scores under 24 on the Mini-Mental Statement Examination [37], or who were assessed as having dementia on study entry, were excluded.

The final baseline sample comprised 1037 participants who met the inclusion criteria. More detailed methods of recruitment and baseline demographics have been previously published [36].

Participants underwent extensive assessments every 2 years, called a “Wave”, during which they completed face-to-face interviews to obtain medical and lifestyle history, a brief medical examination, comprehensive neuropsychological testing, and fasted early morning blood testing. Full details of the neuropsychological and blood testing are described by Trollor et al. [7]. By Wave 2, 889 participants remained in the MAS sample and underwent the same testing procedure as described for baseline. All participants provided written consent to participate in this study, which was approved by the University of New South Wales Human Ethics Review Committee (HC 05037, 09382, 14327).

**Fig. 1:**
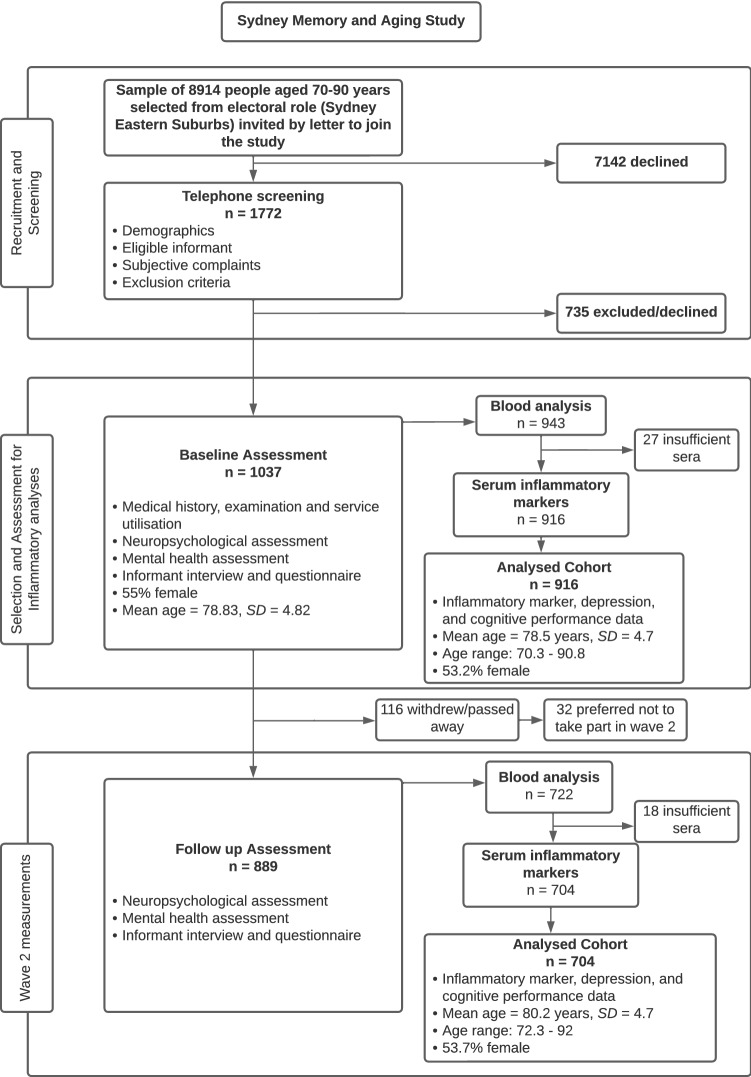
Cohort selection and assessment

We excluded 121 participants without full blood analysis at baseline. In Wave 2, a further 185 participants were excluded due to either missing or insufficient blood analysis. Figure 1 shows the mean age of our participants in Waves 1 and 2. There were no significant difference in the ages of females and males at either wave. Participant characteristics for Waves 1 and 2 are presented in Supplementary Table S1.

#### Cohort selection and assessment

See Fig. 1.

### Measures

#### Bloods

As outlined in Trollor et al. [7], blood was collected after an overnight fast, clotted, aliquoted and frozen at − 80 °C. An array of inflammatory biomarkers was analysed, including IL1β, IL6, IL8, IL10, IL12p70, and CRP. See Supplementary Table S2 for further details of measures.

#### Depression

Depression was measured using the Geriatric Depression Scale [38]. The 15 item self-rated scale calculates a depression score by summing up points allocated to a ‘yes’ or ‘no’ answer. The scale has been proven reliable and valid in the assessment of depressive symptoms in older persons [39].

#### Cognition

As outlined by Trollor et al. [7], a comprehensive neuropsychological test battery was administered. Ten tests were conducted representing the diverse array of cognitive functions impaired with aging (see Table S2). A global cognition score was calculated based on composite domain scores. Domain scores were standardized as z-scores calculated using the mean and standard deviations of a normative reference group at baseline, with higher *z*-scores representing better performance.

#### Somatic, genetic, and lifestyle risk factors

Highest education level achieved was self-reported on a scale from 1 to 5 with 1 being defined as completed primary school, 2 as incomplete high school, 3 as completed high school, 4 as incomplete tertiary education, and 5 as completed tertiary education.

Alcohol consumption was self-reported based on the number of drinking occasions in the last year on a scale from 1 to 6 with 1 being defined as not in the last year, 2 as monthly or less, 3 as 2–4 times a month, 4 as 2–3 times a week, 5 as 4–6 times a week, and 6 as daily.

Cardiovascular disease risk was computed based on the research of the Framingham Stroke Study (see Table S2); specifically, the 10-year risk prediction of general cardiovascular disease from this study [40].

As outlined by Trollor et al. [7], 99% of participants had data relating to APOE genotype. Genomic DNA was extracted from peripheral blood leukocytes using standard procedures and stored at Genetics Repositories Australia (see Table S2). In analysis, participants were coded as either carriers or non-carriers of the ε4 allele.

### Network analyses

The data analysis for this study was approved by the Human Research Ethics Committee of The University of Waikato (NZ). The study was carried out by exploring network associations using baseline data, with Wave 2 data then used to test hypotheses derived through the baseline exploratory analyses. Five inflammatory biomarkers (IL6, IL8, IL10, IL12 and CRP), depression, cognition and other risk factors including education, alcohol consumption, cardiovascular disease risk and presence of APOE-ε4 were included as nodes in the Gaussian graphical model (GGM). Missing values were imputed with the respective posterior predictive distribution given the observed data, as the model was being estimated [41].

#### Estimation and plotting

R software (version 4.0.4) [42] was used to conduct the network analysis, specifically the Bayesian Gaussian graphical models (BGGM) package based on Bayesian estimation because biomarker scores were non-normal and zero inflated [41]. Relationships between nodes in BGGM represent the posterior means of the associations [43]. The “explore” function within BGGM was used to estimate the pairwise relations (i.e., partial correlations) among nodes while accounting for all other relations in the full set of nodes using a semi-parametric copula model based on ranked likelihood. A selected partial correlation matrix was produced which retains an estimate of only those relationships whose 95% credible intervals (CI) did not include zero. A 95% CI indicates a range where a population parameter will fall 95% of the time.

Separate GGMs were estimated in males and females. The “qgraph” function from the R library was used to plot the networks based on the Fruchterman–Reingold algorithm, and the layout was averaged across the GGMs to facilitate comparisons [44]. This algorithm places nodes with the most and strongest edges centrally in the diagram, and pairs of nodes with strong edges close together whilst minimizing overlap [45]. Nodes without edges to the rest of the network represent non-significant associations only, rather than no association. However, the layout of the network should not be interpreted merely on the position of nodes and edges, as these can be unstable [20]. After plotting the network, further analyses were required to test the differences in associations between males and females.

#### Statistical inference and hypothesis testing

To statistically examine whether differences were present, we estimated the mean difference for each node-to-node association in males and females and the 95% CI for this difference using 5000 posterior estimates. 95% CIs of the mean difference estimates that did not contain zero were deemed to represent statistically significant differences. We then formed hypotheses based on significant results and tested these in a confirmatory fashion using Wave 2 data.

Hypothesis testing was conducted in relation to partial correlation networks based on Wave 2 data. These were estimated similarly to the Wave 1 process using the BGGM “estimate” function in R and plotted using qgraph. Mean differences between males and females were calculated as above. Posterior probabilities (PP) were then calculated to determine how confident we were that our hypotheses were supported, with PPs indicating the likelihood of a pre-specified event happening. Keeping with the 95% probability level, we interpreted one directional PPs above 0.95 as indicative of statistically significant differences.

## Results

### Exploratory analyses

**Fig. 2:**
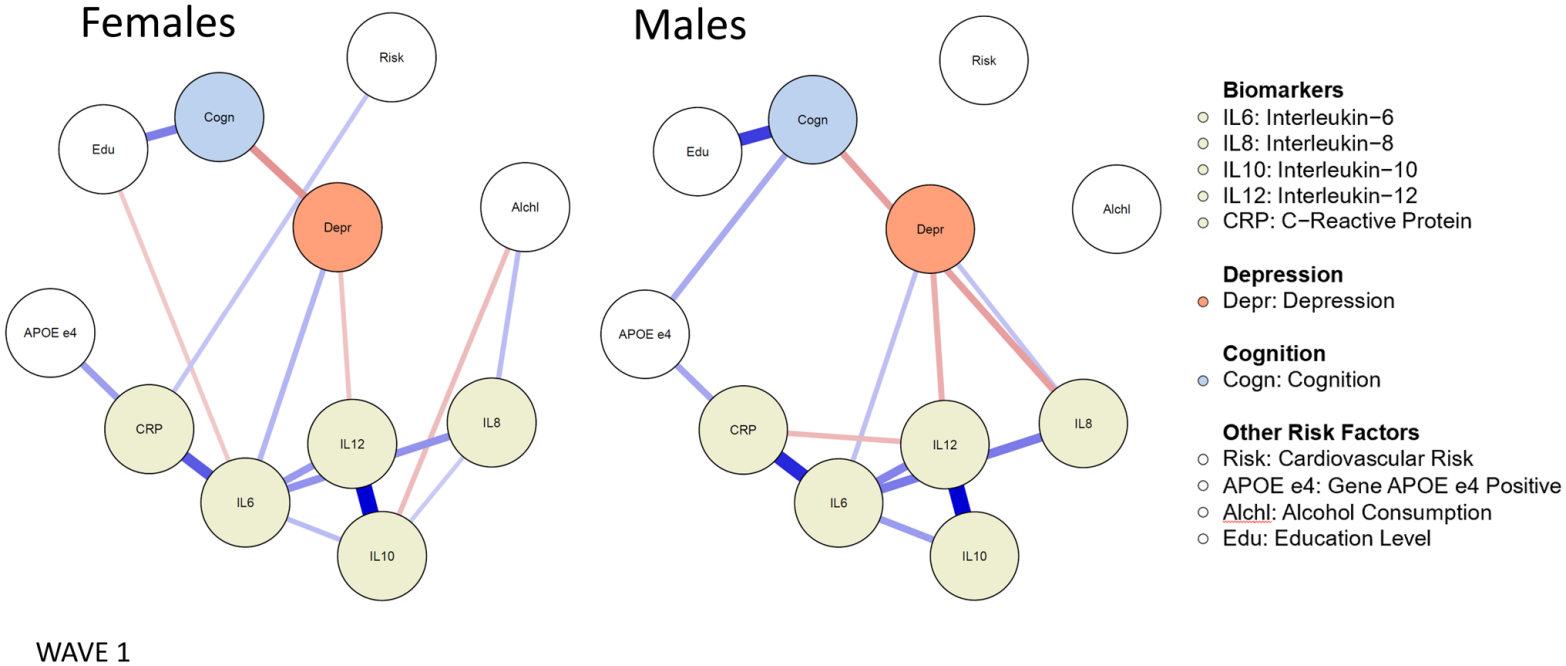
Baseline networks of biomarkers, depression, cognition and other risk factors for females and males at Wave 1. Only associations for which the 95% credible interval did not include zero are printed. “Risk” = Framingham Index cardiovascular disease risk score. Descriptive statistics are available in Supplementary Table S1

Figure 2 shows the networks of unique associations between biomarkers, depression, cognition, and relevant risk factors for females and males at baseline (Wave 1), which are represented by GGMs [46]. Blue edges represent a positive relationship; red edges represent a negative relationship; the thickness and depth of colour in the edges represent the strength of the association with thicker, darker edges representing stronger associations [15].

**Fig. 3:**
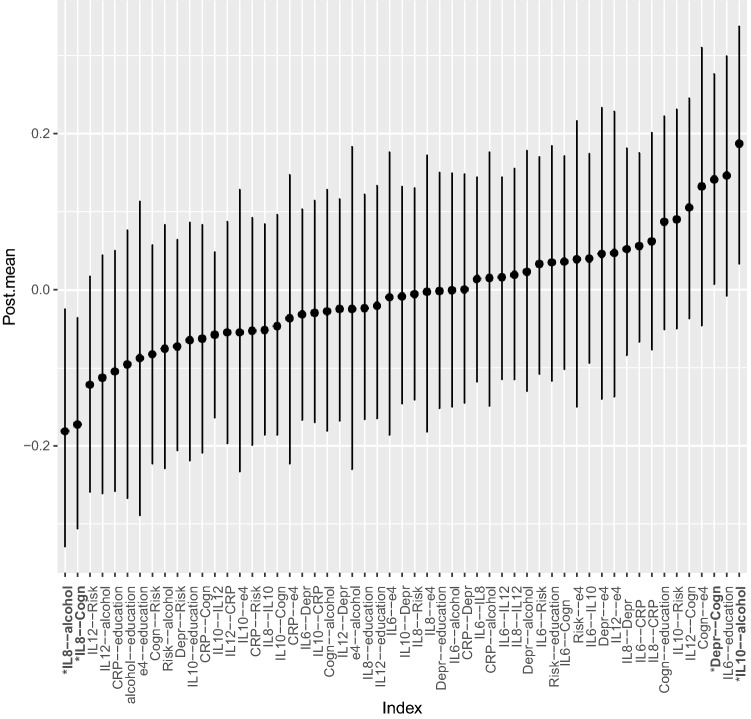
Differences for each node-to-node association between males and females. Dots show mean difference between males and females for each association of Wave 1 data, error bars show 95% CIs for this difference using 5000 posterior estimates. Associations with CIs that do not include zero are in bold with an asterisk and deemed to be statistically significantly different for males and females

Visual inspection of the GGMs highlighted some differences between males and females that were then tested for statistical significance. We estimated the mean difference for each node-to-node association for males and females and the 95% CI for this difference using 5000 posterior estimates. The differences for each node-to-node association between males and females are shown in Fig. 3. Four CIs did not include zero, indicating there was a statistically significant difference in the association between these nodes in males and females. Specifically, depression and cognition were more strongly negatively associated in females; IL8 and cognition were more strongly negatively associated in males; IL8 and alcohol consumption more strongly positively associated in females; and IL10 and alcohol consumption more strongly negatively associated in females. This corresponds with the major differences found when visually inspecting the GGMs. These results emerged from exploratory analyses, which increase the risk of finding false positives. Therefore, we tested these findings as hypothesized relationships using Wave 2 data. Associations that were the same across both males and females included: IL6 positively associated with depression; IL12 negatively associated with depression; education positively associated with cognition; presence of the APOE-ε4 gene positively associated with CRP; and associations between specific biomarkers.

### Confirmatory analyses

**Fig. 4:**
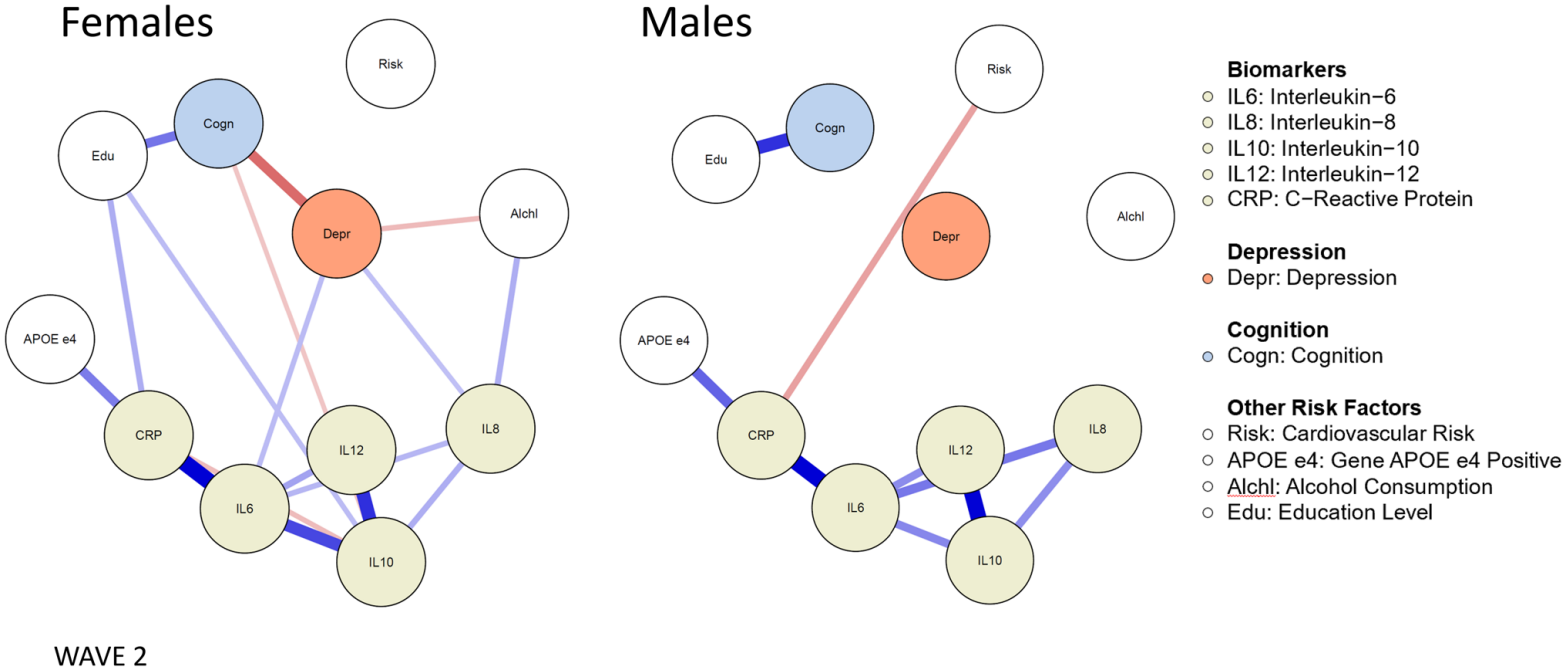
Networks of biomarkers, depression, cognition and other risk factors for females and males at Wave 2. Only associations for which the 95% credible interval did not include zero are printed. “Risk” = Framingham Index cardiovascular disease risk score. Descriptive statistics are available in Supplementary Table S1

To test the hypothesized relationships found in exploratory analysis, we replicated the analysis with a confirmatory sample comprised of follow-up data collected 2 years after baseline. We compared baseline data for participants who completed Wave 2 and those who did not across all demographic variables and found that those who did not complete Wave 2 were significantly older (*M* = 81.6) compared with those who did (*M* = 80.2; *p* < 0.001, *d* = 0.32); those who did not complete Wave 2 had significantly higher depression scores at Wave 1 (mean rank = 511) compared to those who did (mean rank = 438; *p* < 0.001, *d* = 0.32); and those who did not complete Wave 2 had significantly lower cognition scores (*M* = − 0.99) compared with those who did (*M* = − 0.53; *p* < 0.001, *d* = 0.34). There were no significant differences in sex, alcohol consumption, highest education, cardiovascular disease risk, or APOE-ε4 status. Wave 2-based networks are presented in Fig. 4, with a summary of the results presented in Table 1.

**Table 1:** Unique associations at baseline by sex, tested sex differences from baseline data, and confirmed unique associations at Wave 2.

Unique associations between nodes	Exploratory network		Confirmatory network
	Sex baseline	Sex difference	Sex Wave 2
Cognition—depression	F*	M versus F*	F* confirmed
Cognition—IL8	M*	M* versus F	Not confirmed
Cognition—APOE-ε4	M*	M versus F	
Cognition—education	M* and F*	M versus F	M* and F*
Depression—IL6	M* and F*	M versus F	F*
Depression—IL12	M* and F*	M versus F	
Depression—IL8	M*	M versus F	
Alcohol—IL8	F*	M versus F*	F* confirmed
Alcohol—IL10	F*	M versus F*	Not confirmed
Alcohol—depression		M versus F	F*
APOE-ε4—CRP	M* and F*	M versus F	M* and F*
Cardiovascular disease risk—CRP	F*	M versus F	M*
IL6—all other biomarkers	M* and F*	M versus F	M* and F*

*M* = males, *F* = females

*p < .05

Table 1 shows inferential statistics for the relationships of the variables in the network analyses at baseline and Wave 2. We used posterior probability (PP) to calculate the likelihood of associations being stronger based on our hypotheses from baseline. Associations confirmed from baseline to Wave 2 included the negative link between cognition and depression in females (PP females > males = 98%) and the positive link between alcohol consumption and IL8 in females (PP females > males = 97%). Associations not confirmed from baseline to Wave 2 included the negative link found between cognition and IL8 in males (PP males > females = 78%) and the negative link found between alcohol consumption and IL10 in females (PP females > males = 73%). In males, alcohol consumption was not significantly linked to any other variables in the network at either wave.

Although baseline data indicated that IL6 and IL12 were uniquely linked to depression in both males and females, Wave 2 data only confirmed the significant link between IL6 and depression in females. Wave 2 data confirmed the following links from baseline for both males and females: positive association of APOE-ε4 carriers with higher CRP levels; positive association of education and cognition; positive association of IL6 and all other biomarkers. Two new associations in Wave 2 data included a negative association between alcohol and depression in females, and a negative association between CRP and cardiovascular risk in males.

To ensure the robustness of our network findings we also tested differences between variables included in the network between waves using a paired t-test. We found no significant differences between most of the variables with the exception of IL8 and cognition.

Refer to Supplementary Table S3 for details.

## Discussion

The aim of the current study was to investigate networks of inflammatory biomarkers, depression, cognition, and somatic, genetic, and lifestyle risk factors in older males and females. Differences between the male and female networks found during the first assessment were re-examined in the 2-year follow-up data for the purpose of robustness. To ensure the robustness of our network findings, paired t-tests found no differences between most of the variables at the two time points with the exception of IL8 (small effect size) and cognition. Overall cognitive decline with a small effect size is an expected phenomena over time in this age category [47]. This indicates that the relationships can be attributed to the same participants across waves. Our results demonstrated that networks of associations between biomarkers, depression, cognition, and somatic, genetic, and lifestyle factors were significantly different between older males and females.

Specifically, a stable negative link between depression and cognition was found in females, whilst in males, no such significant relationship was evident. A stable positive association between IL6 and depression was found in females, whilst in males this association was not stable. A stable positive association between IL8 and alcohol was found in females, whilst in males this relationship did not reach significance. A stable positive association between APOE-ε4 carriers and CRP was found across males and females. Sex differences in APOE-E4 carriers have been noted regarding the risk for cognitive decline [34], but in our study the effect on cognition is unclear. This could be because our global measure of cognition was not sensitive enough to detect specific differences. A stable positive relationship was also found between education and cognition, as was a positive relationship between IL6 and all other biomarkers. Our measure of cardiovascular disease risk was not significantly different between males and females in our older sample and no stable relationship was found with other variables included in the network. Overall, our results demonstrated significant differences in how inflammatory biomarkers related to mental health and other risk variables in older males and females while the relationship between biomarkers was remarkably similar across sexes.

Our study revealed that the negative relationship between depression and cognition was significantly stronger in older females. A possible mechanism underlying this association could be that depressed females (even older ones) ruminate more than males [48, 49]. Rumination involves repeatedly focusing on depressive symptoms and their meaning and consequences (e.g., “I’m so tired, I won’t be able to finish that job and my boss will fire me”) [49], or mentally rehearsing past stressful events [50]. The resource allocation theory suggests that rumination occupies cognitive resources that subsequently cannot be used for task-oriented challenges [51]. The perseverative cognition hypothesis suggests that rumination causes repeated activation of the stress response, leading to maladaptive physiological responses such as increased inflammation which has been associated with increased depression and declining cognitive function [22, 52].

An implication of our finding that depression and cognition were more strongly negatively linked in older females could be that using interventions to decrease depression in females may also have a protective effect on their cognition. This suggestion seems plausible given that depression is a risk factor for cognitive decline [13]. The timing of any depression-targeting intervention may also be key as earlier life depression has been shown to increase the risk of later cognitive decline [53]. Early intervention may prevent the pathophysiological mechanisms of depression from affecting cognitive performance. Future studies could investigate the contribution of various biopsychosocial strategies in reducing depression and protecting against cognitive decline in older females with the view to tailor the interventions for this population. Future studies might also investigate the directionality of this relationship in experimental and longitudinal studies to inform targeted interventions for depression, specifically in females, which may be most successful if implemented earlier in life.

The positive relationship found between inflammatory biomarker IL-6 and depression indicates that inflammaging may have an association with depression in older females, as also found in the study by Niles et al. [11]. Future experimental studies could investigate whether reducing the levels of this biomarker specifically may reduce depressive symptoms, which may in turn be protective against steeper cognitive decline [54, 55]. In older males, Wave 2 data revealed that depression was not significantly associated with any other variables in the network; thus, inflammation and depression appear not to have a robust significant relationship in males, at least in our sample, and therefore trying to reduce inflammation to reduce depressive symptoms may be more relevant for females. Our findings suggest that trials examining the efficacy of anti-inflammatory drugs on mental health in older individuals should consider possible sex differences in response profiles.

The positive association found between IL-8 and higher alcohol consumption in older females indicates that alcohol may be especially inflammatory in females. This finding reflects other studies, which have found that the effects of alcohol (including on cognition) are more profound in females due to differing body composition [33]. Future longitudinal studies could investigate the directionality of this relationship. In Wave 2, alcohol had a negative relationship with depression in females, indicating that higher levels of alcohol consumption were associated with lower levels of depression. However, this association was not present in Wave 1. Studies have found that a moderate amount of alcohol can protect against depressive symptoms in older people, with abstainers and heavy drinkers suffering significantly higher depressive symptoms compared to moderate drinkers [56]. Moderate amounts of alcohol consumption have been associated with improved quality of life and mood in older people, which could be due to positive effects on cardiovascular health, stress relief, or social support/networking benefits [57]. Future research could investigate this relationship further with a focus on older females.

Whilst Trollor et al. [7] found a significant negative association between IL-12 and the cognitive domain of “processing speed” in older females, with one exception, our study did not find any direct association between inflammatory biomarkers and cognition. At Wave 1, there was a negative association between IL-8 and cognition in males, indicating that higher levels of IL-8 (inflammation) were associated with lower levels of cognition, but this was not replicated at Wave 2. However, our results were based on a composite global cognition factor and did not explore individual domain scores and their relationships with various markers of inflammation. An earlier study by Baune et al. [10] found that IL-8 was associated specifically with poorer memory, cognitive speed, and motor function domains of cognition. IL-8 is a pro-inflammatory cytokine that has been implicated in neurodegenerative and neuropsychological processes in the brain [10]. Our data partially support this implication.

Although cognition was not significantly related to any biomarkers at Wave 2, it was related to education level in both males and females, indicating that older people with higher levels of education scored better on a composite global cognition measure regardless of their sex. This reflects previous research indicating there are no sex differences in the education-cognition relationship [32] and was one of the only significant relationships in our model that was stable across males and females at both waves. The cognition-education relationship is well supported by research [28].

## Strengths and limitations

Whilst an independent sample would be preferred to confirm our exploratory findings, our results are still likely to be generalisable because they were replicated in a subsequent wave. Further research could test our findings against an independent sample. Although the Wave 2 validation provides strong support for the results, a single network analysis method may not be sufficient to fully support the findings of the study. Future studies should replicate the network analysis using other methods and compare the obtained associations among biomarkers with our results. Future experimental and longitudinal studies should also test the causality and directionality of the associations we found to be significant. The use of a global cognitive measure may not have been sensitive enough to pick up specific differences.

The study was conducted with participants recruited from a relatively small catchment area in Sydney, Australia and may not be representative of the wider community. Participants belonged to a predominantly White (European) ethnic group and as such, replicating these analyses on samples comprising other ethnicities would be beneficial. Our study has proposed new research avenues for experimental studies to further examine the relationship between depression and cognition, inflammation and depression, and inflammation and alcohol while accounting for biological sex.

## Conclusion

Our findings suggest partly different psychophysiological mechanisms underlie the interactive networks of biomarkers, depression, and cognition in older males and females and that biological sex should be considered when designing preventive interventions to optimize mentally healthy aging. Inflammaging is likely to be associated with depression in older females.

## Supplementary Information

Below is the link to the electronic supplementary material.

Supplementary file1 (PDF 193 kb)

## Data Availability

The terms of consent for research participation stipulate that an individual’s data can only be shared outside of the MAS investigators group if the group has reviewed and approved the proposed secondary use of the data. This consent applies regardless of whether data has been de-identified. Access is mediated via a standardised request process managed by the CHeBA Research Bank, who can be contacted at ChebaData@unsw.edu.au or via the corresponding author’s contact details at oleg.medvedev@waikato.ac.nz.
