# Supplementary material for: Networks of inflammation, depression, and cognition in aging males and females
Source: Aging Clin Exp Res. 2022 Jul 27;34(10):2387–98. doi: 10.1007/s40520-022-02198-6 (PMC9637618; doi:10.1007/s40520-022-02198-6)
Supplement: Supplementary file 1 — Supplementary file1 (PDF 193 kb) [file 40520_2022_2198_MOESM1_ESM.pdf]

Supplementary Table S1

Descriptive Statistics for the Total Sample and Split by Males and Females (n = 916)

Baseline/Wave 1	Mean	*SD*	Mean - M / mean rank	*SD* - M	Mean - F / mean rank	*SD* - F	sig diff M vs F
IL6	6.56	8.33	6.88	5.67	6.29	10.10	p <.001*
IL8	20.11	13.38	20.15	14.00	20.08	12.80	p = .92
IL10	2.64	1.98	2.55	1.42	2.72	2.37	p = .93
IL12	3.10	2.52	3.03	2.02	3.16	2.89	p = .76
CRP	3.05	5.50	2.94	5.80	3.16	5.23	p = .11
Depression	2.24	2.09	476		441		p = .04*
Cognition	-0.66	1.35	-0.67	1.37	-0.64	1.34	p = .74
Wave 2							
IL6	6.43	8.52	6.65	5.26	6.24	10.60	p < .001*
IL8	19.45	11.56	19.10	10.87	19.80	12.10	p = .23
IL10	2.64	1.97	2.60	1.46	2.68	2.33	p - .63
IL12	3.16	2.34	3.14	2.10	3.19	2.54	p = .003*
CRP	2.82	4.75	2.70	5.36	2.94	4.14	p = .61
Depression	2.06	1.91	400		385		p = .35
Cognition	-0.53	1.31	-0.55	1.30	-0.52	1.32	p = .10
**Other risk factors**	**Median**	**Mean (*SD*)**	***Mdn* / % - M**	**Mean (*SD*) - M**	***Mdn* / % - F**	**Mean (*SD*) - F**	**sig diff**
Alcohol consumption	4.00		5.00		3.00		p < .001*
Highest education	3.00		4.00		3.00		p < .001*
Cardiovascular disease risk		4.1 (3.1)		4.15 (2.6)		4.05 (3.4)	p = .64
APOE-ε4 carrier			23%		23%		p = .93

Note: M = males, F = females; * indicates a significant difference between males and females

Supplementary Table S2

Further details of measures collected

Measure	How measured
CRP	Near Infrared Particle Immunoassay rate methodology using Beckman Coulter Synchron LXi (Beckman Coulter, USA)
Cytokines	The cytokines IL-1β, IL-6, IL-8, IL-10, and IL-12 concentrations were measured using cytometric bead array (CBA, BD Biosciences, San Diego, CA, USA). Six bead populations with distinct fluorescence intensities were coated with capture antibodies specific for the corresponding proteins. These bead populations were mixed to form the BD CBA, which resolved in the FL3 channel of a flow cytometer (BD FACSCalibur). The capture beads, PE-conjugated detection antibodies and recombinant standards were incubated together to form sandwich complexes. Following acquisition of sample data using the flow cytometer, the results were generated in graphical and tabular format using the BD CBA Analysis Software. The intra-assay coefficients of variation were 4–7% for IL-1 β, 5–8% for IL-6, 2–5% for IL-8, 5–6% for IL-10, and 3–6% for IL-12. The inter-assay coefficients of variation were 8–13% for IL-1β, 8–10% for IL-6, 4–7% for IL-8, 8–11% for IL-10, and 6–9% for IL-12.
Cognitive Measures	
Memory	Logical Memory Story A (delayed recall) [58], Rey Auditory Verbal Learning Test (total learning, short-term and long-term delayed recall) [59] and Benton Visual Retention Test recognition [59].
Executive Function	Trail Making Test B [59], and a Controlled Oral Word Association Test [59].
Language	30-item Boston Naming Test [60] and semantic fluency (Animal Naming Task) [59].
Processing Speed	Trail Making Test A [59] and Digit–Symbol Coding task [58].
Visuo-spatial	The Block Design task [58].
Other Measures	
APOE-ε4	APOE genotyping was undertaken by genotyping the two single nucleotide polymorphisms (SNPs, rs7412 and rs429358) that distinguish between the three APOE alleles ε2, ε3 and ε4. Genotyping was performed using Taqman assays (Applied Biosystems Inc. [ABI], Foster City, CA, USA). The validity of the APOE genotyping was confirmed in a subsample using an alternate genotyping method [61]. APOE genotyping results were available for more than 99% of the DNA samples and the allele frequencies in Caucasians for each of the two SNPs were in Hardy-Weinberg equilibrium ($p>0.05$).
Cardiovascular disease risk	Framingham index: current smoking status, diabetic status, systolic blood pressure, total cholesterol level, high-density lipoprotein (HDL) level, and if currently taking antihypertensive medication. If blood analysis was unavailable, Body Mass Index was used instead of cholesterol and HDL data. In analysis, a risk score was calculated by allocating points to each risk factor. The score reflects the additive impact of specific health factors on the risk of developing cardiovascular disease over a 10-yr period (where the greatest risk is the main effect of age and sex).

Note: References [58 - 61] are found in the main reference list of the article.

Supplementary Table S3

Descriptive Statistics Compared Across Waves 1 and 2 (Paired t-tests; n =704)

	Baseline/Wave 1		Wave 2			
	Mean	*SD*	Mean	*SD*	*t*-stat	sig diff
IL6	6.43	8.52	7.47	19.82	-1.50	$p = .13$
IL8	19.45	11.56	17.87	9.98	3.50	$p < .001$*
IL10	2.64	1.97	3.60	14.18	-1.88	$p = .06$
IL12	3.16	2.34	3.23	30.24	-0.07	$p = .95$
CRP	2.80	4.77	3.11	8.19	-0.89	$p = .37$
Depression	2.17	1.99	2.35	1.32	-2.57	$p = .01$
Cognition	-0.57	1.32	-0.82	1.50	9.94	$p < .001$*

Note. p-value compared to .007 due to Bonferroni correction for 7 t-tests.

* indicates a significant difference between two time points
